# Loss of tumor suppressor menin expression in high grade cholangiocarcinomas

**DOI:** 10.1186/s13104-023-06282-6

**Published:** 2023-02-13

**Authors:** Terry C. Lairmore, Jehan Abdulsattar, Arrigo De Benedetti, Runhua Shi, Shile Huang, Md Imtiaz Khalil, Stephan N. Witt

**Affiliations:** 1 Department of Surgery, Louisiana State University Health Sciences Center, Shreveport, LA 71103 USA; 2 Feist-Weiller Cancer Center, Louisiana State University Health Sciences Center, Shreveport, LA 71103 USA; 3 Department of Pathology, Louisiana State University Health Sciences Center, Shreveport, LA 71103 USA; 4 Department of Biochemistry and Molecular Biology, Louisiana State University Health Sciences Center, Shreveport, LA 71103 USA

**Keywords:** Cholangiocarcinoma, Fibrosis, Menin, Tumor suppressor

## Abstract

**Background:**

*MEN1*, which codes for the protein menin, is a tumor suppressor in neuroendocrine tissue. In cholangiocarcinoma (CCA) cell lines the overexpression of menin decreased proliferation, angiogenesis, migration, and invasion in vitro and in xenografts, but its expression in CCA tumor tissue samples is not established.

**Objective:**

Determine whether the expression of menin correlates with disease progression in patient samples of CCA in a tissue microarray (TMA) by immunohistochemical (IHC) staining.

**Results:**

IHC analysis of 97 biopsies revealed that low-grade tumors (Grade I) exhibited intense, diffuse, finely granular nuclear menin immunoreactivity with a pronounced linear perinuclear pattern (mean IHC score = 2.00), whereas high-grade tumors (Grade III) mostly lacked such staining (mean IHC score = 0.35). Collectively, there was a significant inverse association between tumor grade and menin staining (*P* = 0.0005). We also found a significant association between fibrosis status and menin staining, in that, 81.2% (56/69) of patients without fibrosis had no menin staining, whereas 92.9% (26/28) patients with fibrosis exhibited menin staining (*P* < 0.0001). No association was found between fibrosis status and grade. Overall, menin expression is inversely associated with tumor grade and positively associated with fibrosis status.

**Supplementary Information:**

The online version contains supplementary material available at 10.1186/s13104-023-06282-6.

## Background

Cholangiocarcinoma (CCA) is an aggressive primary liver tumor that arises from the cells lining the bile ducts. The incidence of CCA has been increasing worldwide for the past 30 years, with approximately 8000 cases per year in the United States. CCA accounts for approximately 15% of all primary liver cancers and 3% of gastrointestinal malignancies and has a very aggressive disease course and poor prognosis overall. Despite the rare occurrence of CCA, it accounts for approximately 2% of all cancer-related deaths worldwide due to its refractoriness to chemotherapy, and often insidious presentation accounting for frequent diagnosis at a late stage when the tumor is not surgically removable or curable [1]. Further, there are few molecular targets that have been identified for effective therapies. Given the aggressive nature of CCA and the limited responsiveness to current available therapies, there is a need to further elucidate the cellular oncogenic pathways involved in CCA progression, providing expanded opportunities for molecular targeting.

The *MEN1* tumor suppressor gene encodes menin, a nuclear protein that has been shown to function as a scaffold protein involved in broad control of transcriptional regulation, including important epigenetic effects. Menin acts as a tumor suppressor in endocrine tissues by regulating several important signaling pathways by controlling gene transcription but has an oncogenic role in leukemic transformation through alternate interactions with the mixed lineage leukemia complex [2–4]. Loss of menin function has been associated with the development of diverse cancer types, not only endocrine tissue derived neoplasms, but also hepatocellular carcinoma (through epigenetic up-regulation of Yap1 transcription) [5], melanoma [6], and breast cancer [7]. This emphasizes a broader role for menin in the regulation of gene transcription that affects many downstream signaling pathways, by interacting with partner proteins involved in vital cell activities such as DNA damage repair, cell division, cell proliferation, and genome stability.

Previous studies in a variety of CCA cell lines demonstrated that menin expression was decreased in advanced CCA, whereas expression of microRNA-24 (miR-24) was inversely related and increased in advanced CCA [8]. These studies investigated CCA cell lines and tumor xenografts after menin/miR-24 manipulation. Menin overexpression decreased cell proliferation, migration, invasion, and angiogenesis. Inhibition of miR-24 increased menin protein expression while decreasing cell proliferation, migration, invasion, and angiogenesis [8]. Epigenetic factors, such as the post-translational modification of menin by microRNAs, are believed to have a pivotal role in tissue dependent drive for tumor formation. The role of menin and the reciprocal role of the microRNA miR-R24 [9] in CCA represents a potential novel pivotal pathway for CCA development which may allow identification of targeted therapeutic approaches. We sought to determine the expression of menin in human CCA tumor samples, and to correlate menin expression with tumor grade as well as other tumor characteristics including associated fibrosis.

## Materials and methods

### Reagents

The CCA TMA (#LV1004a) was purchased from US Biomax, Inc (Derwood, MD, USA); as of 2023, this product is distributed by TissueArray.com. The company (US Biomax) that generated this CCA tissue microarray obtained donor consent and all tissue was collected under HIPPA approved protocols (see https://www.tissuearray.com/FAQs). Rabbit monoclonal anti-menin antibody (#ab92443) was obtained from Abcam (Cambridge, MA, USA). Both VECTASTAIN Elite ABC-HRP Kit (Peroxidase, Universal, #PK-6200) and ImmPACT DAB Substrate, Peroxidase (HRP) (#SK-4105) were purchased from Vector Laboratories (Newark, CA, USA).

### Immunohistochemistry

The sections with TMAs were deparaffinized and rehydrated following a standard protocol. Next, the TMAs were incubated with anti-menin antibody (#ab92443) at 1:50 dilution, at 36 °C for 1 h. Sections were washed with PBS and the antigen-antibody complexes were further stained using Vectastain Elite ABC Reagent (Vector Laboratories). Finally, the sections were developed using DAB substrate (Vector Laboratories) for visualization. A section was counterstained with hematoxylin and eosin (H&E). All sections were mounted with di-n-butylphthalate-polystyrenexylene (DPX) and the slides were visualized with a BX41 Olympus inverted microscope equipped with a digital camera (Olympus DP71). Images were acquired at 20X magnification. Menin staining was scored as follows: no staining = 0, very weak and patchy perinuclear immunoreactivity = 1, moderately intense perinuclear linear immunoreactivity = 2, and intense, diffuse, and finely granular nuclear immunoreactivity with a more pronounced linear perinuclear pattern = 3. The fibrosis was identified and considered in this project when the tumor glands were embedded in more than 60% of a dense collagenous glassy fibrotic background. We also graded tumors from I to III, in accordance with recommendations from the College of American Pathologists.

### Bioinformatics analysis

The *MEN1* mRNA transcript levels versus tumor grades were generated by interrogating The Cancer Genome Atlas-Cholangiocarcinoma (TCGA-CCA) datasets using The University of Alabama at Birmingham Cancer data analysis portal (accessed on October 20, 2022). Survival analysis of cholangiocarcinoma patients from TCGA-CCA dataset based on high or low *MEN1* transcript level was generated using Gene Expression Profiling Interactive Analysis 2 online tool (GEPIA) with a high cut-off value of 70% and low cut-off value of 30% (accessed on October 20, 2022).

### Statistical analysis

Statistical Software SAS for Windows 9.4 (SAS Institute Inc., Cary, NC) was used for all the statistical analyses. The Fisher exact test was used for the association analyses. The Kruskal-Wallis test was used to assess differences between menin staining (IHC scores) versus tumor grade in the violin plot. For each test, a *P*-value less than 0.05 was considered as statistically significant. GraphPad Prism was used to prepare the violin plot.

## Results

We interrogated the TGCA-CCA datasets to gain insight into the variation of *MEN1* mRNA level as a function of tumor grade. Unfortunately, because of the small number of patient samples (N = 36), the plot of *MEN1* transcript level versus grade had no statistically significant differences. Qualitatively, there was a decrease in *MEN1* transcript level going from grade III to IV (Additional file 1: Fig. S1). We also attempted to correlate survival to *MEN1* transcript level using the TCGA-CCA dataset and the GEPIA tools. Although the survival plot shows no statistical significance by the log-rank test, there is a trend toward longer survival for patients with the highest *MEN1* transcript levels (Additional file 1: Fig. S2).

To investigate the expression of menin in CCA tumors, and its association with disease progression, we performed immunohistochemical staining of CCA tumor samples from 97 patients. The clinicopathologic features for the patients corresponding to the tumor samples in the study are given in Additional file 2: Table S1. 29% of the biopsies showed evidence of tumor-associated fibrosis. All data is in Additional File 3.

**Fig. 1:**
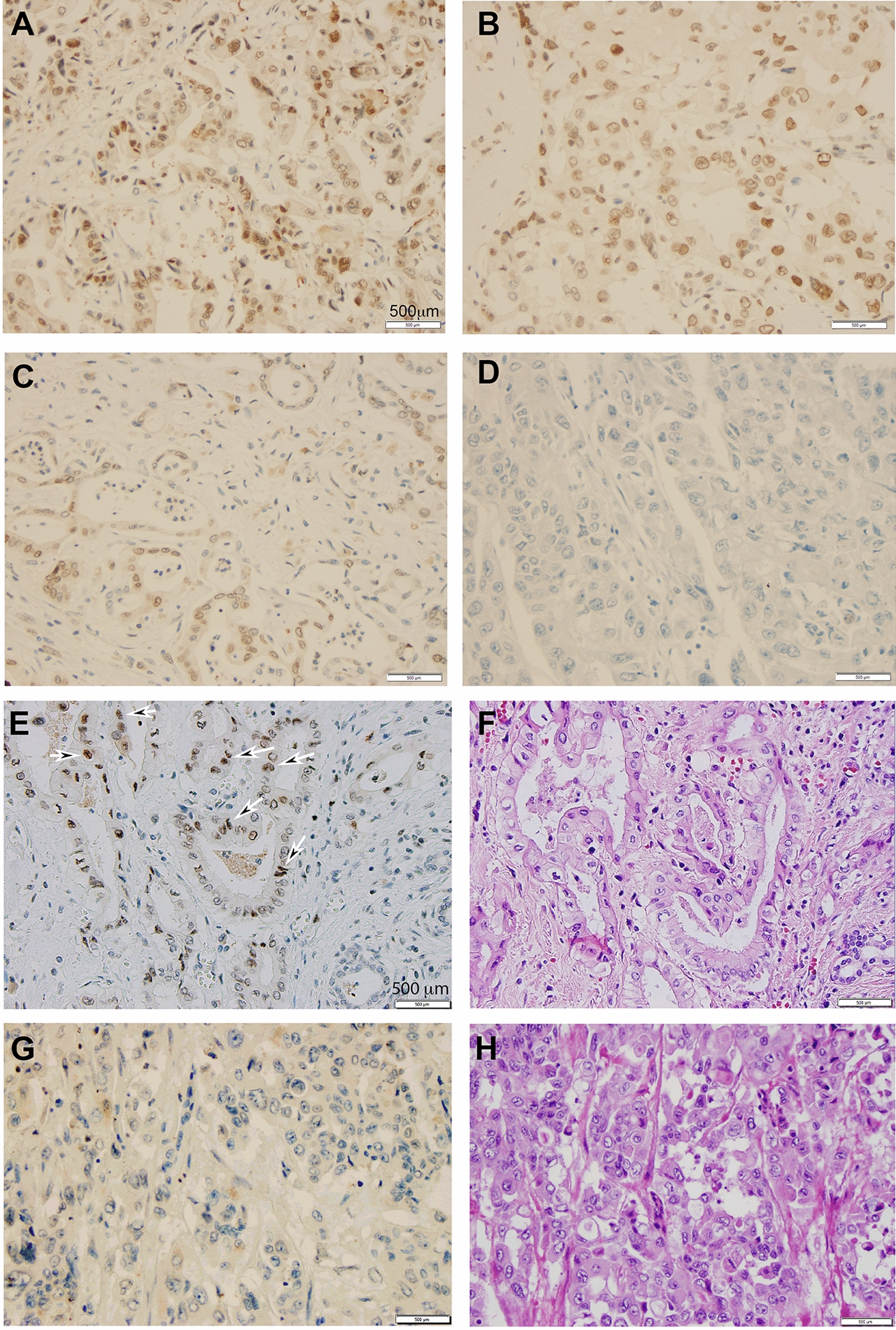
The expression level of menin is inversely related to the grade of the CCA. The images in this figure were obtained from the immunohistochemical staining of CCA tumors in the microarray. Immunohistochemical staining of menin showing the range of IHC scores. **A** Menin IHC = 3, grade I. **B** Menin IHC = 2, grade I. **C** Menin IHC = 1, grade II. **D** Menin IHC = 0, grade III. Immunohistochemical staining of menin and H&E staining. **E**, **F** Menin IHC = 3, Grade I. **G**, **H** Menin IHC = 0, Grade III. Arrows indicate menin-immunoreactivity. Bar = 500 μm

Because immunostaining of CCA tumor cores for menin expression was the basis for this study, the range of menin immunostaining intensities are given in Fig. 1A–D. Panels A and B show menin IHC scores of 3 and 2, respectively. (Each of these samples are grade I tumors.) Panels C and D show menin IHC scores of 1 and 0, respectively. (Panel C is a grade II tumor and panel D is grade III.)

In Fig. 1E–H, we have added H&E staining which reveals information on the morphological features of the tissue. In these images we compared low- and high-grade tumors. Representative images of Grade I tumor cores immunostained for menin and with H&E staining are shown in Fig. 1E, F, respectively. In this low-grade tumor, the menin staining pattern (IHC score = 3) appeared as an intense, finely granular diffuse nuclear positivity with a pronounced linear perinuclear pattern. (Fig. 1E). The corresponding H&E staining shows a well-differentiated CCA in which the malignant cells are arranged in haphazard glandular patterns (Fig. 1F). Collectively, 90% (9/10) Grade I CCA cores had menin IHC scores greater or equal to one (Table 1D).

**Table 1:** Association between menin and clinicopathological features

	Menin staining (IHC score)								Total	*P*-value^a^
	0		1		2		3			
A										
Age (years)	n	%	n	%	n	%	n	%	n	
≤ 50	18	56.25	3	9.38	7	21.88	4	12.5	32	0.1504
> 50	40	61.54	15	23.1	6	9.23	4	6.15	65	
B										
Sex										
F	25	59.52	7	16.7	8	19.05	2	4.76	42	0.4114
M	33	60	11	20	5	9.09	6	10.91	55	
C										
AJCC Stage										
II	22	52.38	6	14.3	8	19.05	6	14.29	42	0.3206
III	14	63.64	6	27.3	1	4.55	1	4.55	22	
IVA	22	66.67	6	18.2	4	12.12	1	3.03	33	
D										
Grade	n	%	n	%	n	%	n	%	n	
I	1	10	2	20	3	30	4	40	10	0.0005
II	25	56.8	8	18	8	18.2	3	6.8	44	
III	32	74.4	8	19	2	4.7	1	2.3	43	
E										
Fibrosis										
N	56	81.2	7	10	2	2.9	4	5.8	69	< .0001
Y	2	7.1	11	39	11	39.3	4	14.3	28	
F										
Grade	Patients without fibrosis									
I	1	12.5	2	25	2	25	3	37.5	8	< .0001
II	24	85.7	3	10.7	0	0	1	3.6	28	
III	31	93.9	2	6.1	0	0	0	0	33	
G										
Grade	Patients with fibrosis									
I	0	0	0	0	1	50	1	50	2	0.3829
II	1	6.3	5	31	8	50	2	12.5	16	
III	1	10	6	60	2	20	1	10	10	

^a^Fisher exact test

Representative images of Grade III tumor cores immunostained for menin and with H&E staining are shown in Fig. 1G, H, respectively. In this high-grade, poorly differentiated tumor, menin immunoreactivity was absent (IHC score = 0) (Fig. 1G). The corresponding H&E staining shows a poorly differentiated CCA in which the tumor cells have an enlarged and pleomorphic nucleus with a high nuclear-to-cytoplasmic ratio, and the malignant cells are arranged mostly in glandular and diffuse patterns (Fig. 1H). Collectively, 32 of the 43 (74%) Grade III CCA cores had menin IHC scores equal to zero (Table 1D).

A Fisher exact test was used to test whether there were statistically significant associations between the attributes age, sex, tumor stage, tumor grade, or fibrosis status and menin expression (IHC score) (Table 1). First, we found no association between age or sex and menin expression (Table 1A, B). Second, we found no association between clinical stage (the extent to which to the tumor has grown and spread) and menin expression (Table 1C). Third, we tested for an association between tumor grade and menin expression using data from all biopsies (non-fibrotic cores and fibrotic). For this group, there was a statistically significant inverse association between grade and menin expression, i.e., the higher the tumor grade the lower the menin staining (Table 1D; *P* = 0.0005). Fourth, we found a statistically significant association between the fibrosis status (Y, N) and menin expression (Table 1E; *P* < 0.0001). For example, 81.2% (56/69) of patients without fibrosis had no menin staining (IHC score = 0), whereas 92.9% (26/28) patients with fibrosis exhibited appreciable menin staining (IHC score ≥ 1). Fifth, a possible association between tumor grade and menin expression was assessed by examining fibrosis status. For the 69 cores with no fibrosis, a strong statistically significant inverse association existed between tumor grade and menin expression (Table 1F; *P* < 0.0001). In contrast, no association was found between tumor grade and menin expression for the 28 cores with a positive fibrosis status (Table 1G; *P* = 0.3829).

**Fig. 2:**
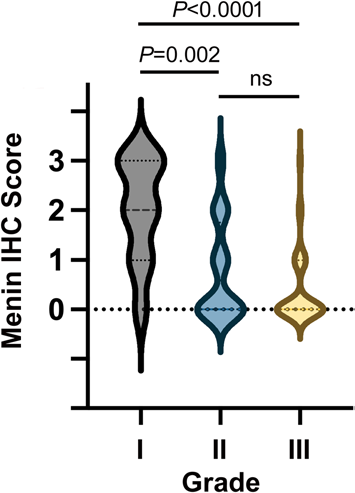
Violin plot of menin IHC scores as a function of tumor grade. Data is taken from Table 1D. Mean menin IHC scores for grades I, II, and III are 2.00, 0.75, and 0.35, respectively. The indicated *P*-values were calculated using a Kruskal-Wallis test. The number of patient samples were N = 10, 44, and 43 for grades I, II, and III, respectively. *ns* not significant

To gain insight into the differences in menin expression (IHC score) and grade, we constructed a violin plot of Menin IHC score versus tumor grade. To determine if the differences between the distributions are statistically significant, we used the Kruskal-Wallis test. Significant differences in the distribution of menin IHC scores were found for grades I and II and grades I and III but not for grades II and III (Fig. 2).

## Discussion

The main finding from our analysis of 97 CCA tumor samples is that lack of menin expression is strongly associated with high tumor grade (*P* = 0.0005). This finding builds upon prior studies using a variety of CCA cell lines, where we showed that overexpressing menin decreased proliferation, angiogenesis, migration, and invasion [8]. Furthermore, the level of the microRNA, miR-24, was found to be inversely related to menin protein level, and inhibiting miR-24 resulted in menin overexpression and decreased proliferation, angiogenesis, migration, and invasion [8]. Finally, in vivo treatment of a CCA xenograft with a miR-24 inhibitor decreased tumor growth while increasing menin expression [8]. In the future, when we gain access to larger volumes of CCA samples, we will probe the tumors for miR-24, and we predict that miR-24 will increase with increasing tumor grade. Overall, we hypothesize that the low level of menin expression in high grade CCAs leads to an upregulation of disease-defining molecular pathways that promote an aggressive phenotype and tumor progression.

Fibrosis is commonly found in liver biopsy samples, and 29% of the CCA cores in the TMA were positive for fibrosis. Unless the tumor areas are carefully dissected out, fibrotic areas may complicate gene expression analyses of CCA in TCGA or other repositories, particularly since CCA is a rare cancer and relatively few sequenced cases have been deposited. It is now well accepted that cancer associated fibroblasts (CAF) form a complex interaction with the tumor microenvironment. Often, they display a pattern of gene expression very different from that of the tumor adenocarcinoma component, although sometimes they also mimic each other [10, 11]. How such a microenvironment affects the behavior of the primary cancer requires much more work to elucidate; it is possible that the interaction between CAF and tumor cells may delay the onset of loss of menin expression in cancer cells, and potentially slow their progression to a more aggressive disease state. Therefore, caution should be used when evaluating CCA biopsies that contain fibrosis.

Our statistical analysis, shown in Table 1, provides evidence of possible interplay between CAF and CCA tumor cells that alters menin expression. For example, a strong inverse association between tumor grade and menin staining was found when all biopsy samples (non-fibrotic and fibrotic) were analyzed (*P* = 0.0005; Table 1D). A similar strong inverse association between tumor grade and menin staining was found when only non-fibrotic biopsies were analyzed (*P* < 0.0001; Table 1F). In contrast, strikingly, there was no statistically relevant association between grade and menin staining when only fibrotic samples were analyzed (*P* = 0.3829; Table 1G). This latter finding suggests that a fibrotic microenvironment affects gene expression in the CCA cells, resulting in a delay in the loss of menin expression. Buttressing this idea, 93.9% of the Grade III tumors without fibrosis exhibited a menin IHC score = 0 (Table 1F), whereas only 10% of the Grade III tumors with fibrosis exhibited a menin IHC score = 0 (Table 1G). The molecular details of how fibrosis could affect menin expression are not known.

In sum, we found a strong, statistically significant inverse association between CCA tumor grade and menin expression in tumor tissue samples. It will be of great interest to decipher the disease-defining pathway(s) that are activated by the loss of menin expression.

## Limitations

The main limitation in this study is small sample size, in some cases. There was a relatively low number (n = 28) of CCA cores that were positive for fibrosis status. Likewise, there was a low number (n = 10) of Grade I CCA cores. On the other hand, given the strong associations and differences revealed in Table 1 and Fig. 2, the authors are confident that the total number of samples distributed among the three grades lead to valid conclusions.

## Supplementary Information

**Additional file 1: Figure S1.** *MEN1* mRNA transcript level as a function of tumor grade. This plot was made using a TCGA-CCA dataset (N=36). There were no statistically significant differences between the transcript levels in the different grades. **Figure S2.** Survival analysis. Survival analysis of TCGA CCA patients based on high or low *MEN1* transcript level. This plot was made using the GEPIA 2 online tool using a high cut-off value = 70% and low cut-off value = 30% (accessed on October 20, 2022).

**Additional file 2: Table S1.** Patient characteristics and clinical presentation. There were 97 patients in this study, and the mean age was 54.5 years old (SD=10.5).

**Additional file 3: **Excel file containing all data acquired in this study.

## Data Availability

All data generated or analyzed during this study are included in this published article (and in its Additional files).

## Abbreviations

CAF: Cancer-associated fibroblast
CCA: Cholangiocarcinoma
GEPIA: Gene Expression Profiling Interactive Analysis 2 online tool
IHC: Immunohistochemistry
*MEN1*: Gene that codes for the tumor suppressor protein menin
TCGA: The cancer genome atlas
TMA: Tissue microarray
