# Supplementary material for: Loss of tumor suppressor menin expression in high grade cholangiocarcinomas
Source: BMC Res Notes. 2023 Feb 13;16:15. doi: 10.1186/s13104-023-06282-6 (PMC9923918; doi:10.1186/s13104-023-06282-6)
Supplement: Supplementary file 1 — Additional file 1: Figure S1. MEN1 mRNA transcript level as a function of tumor grade. This plot was made using a TCGA-CCA dataset (N=36). There were no statistically significant differences between the transcript levels in the different grades. Figure S2. Survival analysis. Survival analysis of TCGA CCA patients based on high or low MEN1 transcript level. This plot was made using the GEPIA 2 online tool using a high cut-off value = 70% and low cut-off value = 30% (accessed on October 20, 2022). [file 13104_2023_6282_MOESM1_ESM.docx]

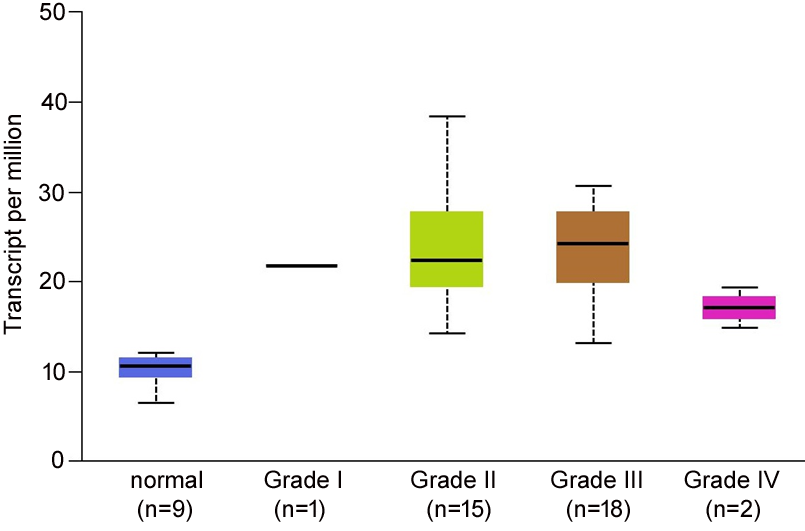


Figure S1: *MEN1* mRNA transcript level as a function of tumor grade. This plot was made using a TCGA-CCA dataset (N=36). There were no statistically significant differences between the transcript levels in the different grades.


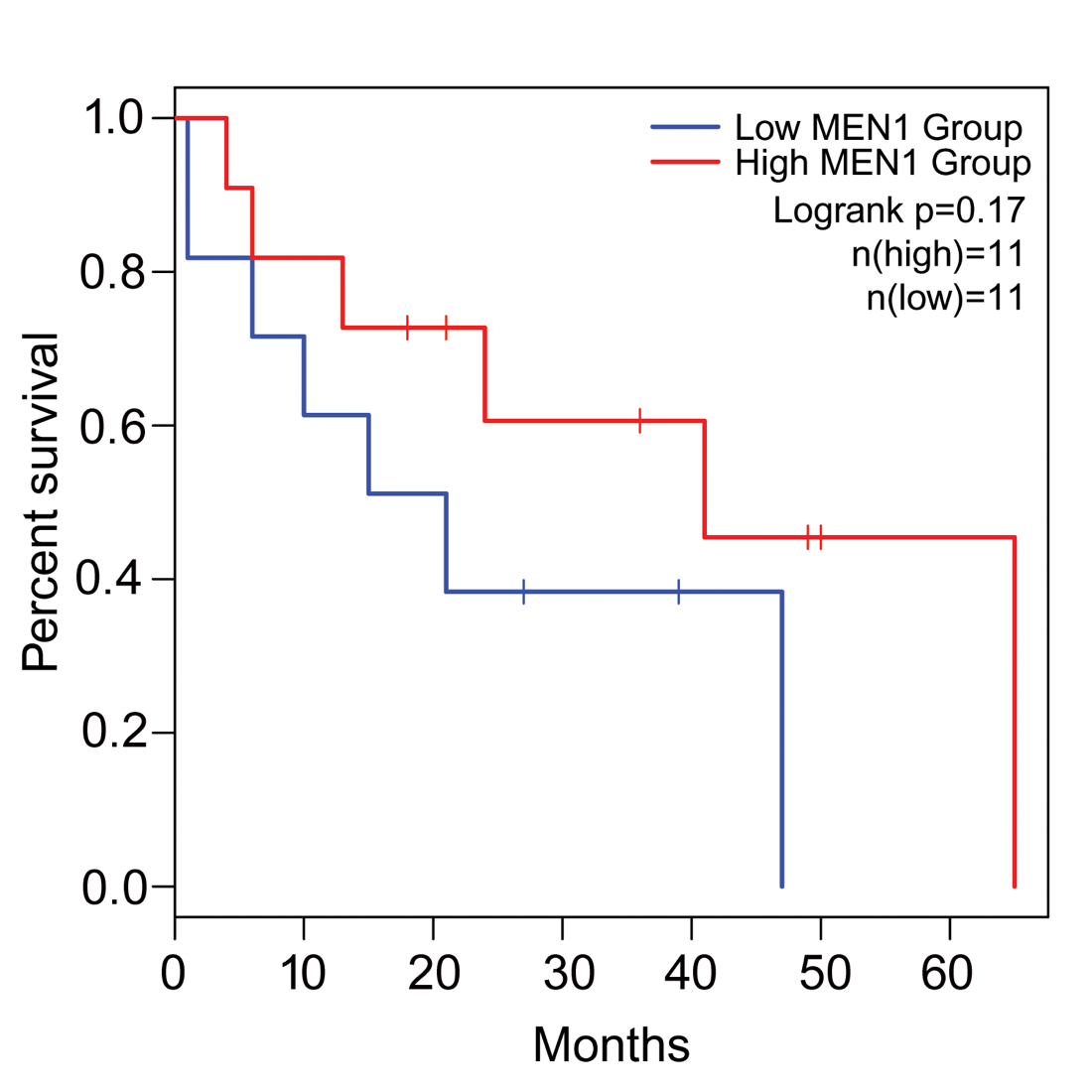


Figure S2: Survival analysis. Survival analysis of TCGA CCA patients based on high or low *MEN1* transcript level. This plot was made using the GEPIA 2 online tool using a high cut-off value = 70% and low cut-off value = 30% (accessed on October 20, 2022).
