# Supplementary material for: Loss of tumor suppressor menin expression in high grade cholangiocarcinomas
Source: BMC Res Notes. 2023 Feb 13;16:15. doi: 10.1186/s13104-023-06282-6 (PMC9923918; doi:10.1186/s13104-023-06282-6)
Supplement: Supplementary file 2 — Additional file 2: Table S1. Patient characteristics and clinical presentation. There were 97 patients in this study, and the mean age was 54.5 years old (SD=10.5). [file 13104_2023_6282_MOESM2_ESM.docx]

Table S1: Patient characteristics and clinical presentation. There were 97 patients in this study, and the mean age was 54.5 years old (SD=10.5).

| Factors | Levels | n | % |
| --- | --- | --- | --- |
| Age (years) | **≤50** | 32 | 32.99 |
|  | **>50** | 65 | 67.01 |
| Sex | **F** | 42 | 43.30 |
|  | **M** | 55 | 56.70 |
| Grade | **1** | 10 | 10.31 |
|  | **2** | 44 | 45.36 |
|  | **3** | 43 | 44.33 |
| Fibrosis | **No** | 69 | 71.13 |
|  | **Yes** | 28 | 28.87 |
| Menin Expression | **0** | 58 | 59.79 |
|  | **1** | 18 | 18.56 |
|  | **2** | 13 | 13.4 |
|  | **3** | 8 | 8.25 |
| AJCC stage | **II** | 42 | 43.30 |
|  | **III** | 22 | 22.68 |
|  | **IVA** | 33 | 34.02 |
